# Is Tamoxifen Use a Factor Affecting Continence in Breast Cancer Patients?

**DOI:** 10.7759/cureus.5417

**Published:** 2019-08-18

**Authors:** Goksen Inanc Imamoglu, Tulay Eren, Oguz Arzu, Nuriye Yıldırım, Cengiz Karacin, Burhan Baylan

**Affiliations:** 1 Oncology, Diskapi Yildirim Beyazit Training and Research Hospital, Ankara, TUR; 2 Oncology, Baskent University Faculty of Medicine, Ankara, TUR; 3 Urology, Diskapi Yildirim Beyazit Training and Research Hospital, Ankara, TUR

**Keywords:** breast cancer, tamoxifen, urinary incontinence

## Abstract

Introduction: Tamoxifen treatment has been shown to reduce the recurrence and mortality rates in hormone receptor-positive breast cancers independent from chemotherapy. This benefit increases with the prolongation of the use of tamoxifen but with increasing side effects. In this study, we aim to evaluate the presence of urogenital symptoms in breast cancer patients on tamoxifen and compare them with those who are not on any hormonotherapy.

Materials and methods: This study was performed on patients diagnosed as early-stage breast cancer. The study group consisted of hormone receptor-positive patients given tamoxifen as adjuvant hormonal therapy. The control group consisted of breast cancer patients who had no hormonotherapy. Patients with a complaint of urinary incontinence with onset after tamoxifen usage were evaluated with Urogenital Distress Inventory Short Form (UDI-6), Incontinence Impact Questionnaire Short Form (IIQ-7) and Incontinence Quality of Life Questionnaire (I-QOL).

Results: A total of 137 early-stage breast cancer patients were included in this study; 74 estrogen receptor-positive patients on tamoxifen therapy (study group) and 63 hormone receptor-negative patients with no hormonotherapy (control group). The median age was 44 (30-65) years for tamoxifen users and 49 (27-64) years for the control group. The stages of the patients were similar for both groups. 78.4% of the women in the tamoxifen group and 49.2% in the control group were in the premenopausal period. The groups were similar in regard to body mass index and parity. The complaint of urinary incontinence was more frequent in the study group compared to controls (39 (52.7%) vs. 5 (7.9%)). Women with the complaint of urinary incontinence were evaluated with self-reported UDI-6, IIQ-7 and I-QOL forms and the scores were similar for both study and control groups. A statistically significant relation was observed between cigarette smoking and the presence of urinary incontinence. The percentages of smokers were 50% of those with incontinence and 24.7% of those without incontinence.

Conclusion: Urinary incontinence is positively correlated with tamoxifen usage in early-stage breast cancer patients.

## Introduction

Breast cancer is the most frequently diagnosed cancer in women globally and it is the second leading cause of cancer deaths after lung cancer [1]. With a 43.0/100,000 age-standardized cancer rate, breast cancer is also the leading cancer in women in Turkey [2]. In hormone receptor-positive breast cancer subtype, constituting about 75% of all cases, endocrine treatment has been shown to reduce the recurrence and mortality rates, independent from chemotherapy [3]. Adjuvant endocrine treatment shows its effect via creating estrogen deprivation, either at the receptor level (SERMs) or by blocking its synthesis (aromatase inhibitors); the latter being an option for the postmenopausal group.

Extended five-year follow-up analysis of tamoxifen studies have shown reduced locoregional and distant breast cancer recurrence rates for up to 10 years (approximately 50% lower than that in the control group in the first five years and 30% lower during the next five years) and reduced breast cancer mortality by about one-third throughout 15 years as carry-on effect (p<0.0001) irrespective of age, nodal status, or use of chemotherapy [4]. Long term follow-up data from estrogen receptor-positive breast cancer patients who received adjuvant tamoxifen therapy for five years, has shown that distant recurrences occurred at a steady rate for at least another 15 years after the end of the five-year treatment period, the risk being strongly correlated to tumor and nodal status and varying between 10%-41% [5]. This finding had led to several randomized trials that investigated the effects of extended use of hormonal therapies. In the Adjuvant Tamoxifen: Longer Against Shorter (ATLAS) trial and Adjuvant Tamoxifen Treatment Offer More (aTTom) trial, it was shown that additional five years of tamoxifen (a total treatment period of 10 years) treatment led to improved relapse-free survival (RFS) and overall survival (OS) when compared with five years of tamoxifen treatment [6-7].

However, all this benefit of tamoxifen was at the expense of increased risk of side effects with extended treatment [8]. The side effects of tamoxifen are mostly onset or exacerbation of menopausal symptoms and urogenital side effects that affect the quality of life of the patients [9-10]. Since the female urogenital tract is extremely sensitive to estrogen, low levels caused by hormonotherapies can lead to urogenital symptoms such as vaginal dryness, burning, irritation, urgency, urinary incontinence, and sexual dysfunction. The effect of tamoxifen for urinary incontinence has not been extensively studied and reported, so there is only inconclusive information in the literature. In this cross-sectional study, we aim to evaluate the presence of urogenital symptoms in breast cancer patients on tamoxifen and compare with those who are not on any hormonotherapy to see if tamoxifen has any effect.

## Materials and methods

Patients with a diagnosis of early-stage breast cancer from a single oncology center in Ankara were included in this study. Patients who are still on primary therapy as radiotherapy or chemotherapy; those with a recurrent/metastatic disease; those with a history of urological problems as resistant urinary infections or presence of a complaint of urinary incontinence before the start of hormonotherapy; those with a history of neurologic or musculoskeletal problems and patients who are on aromatase inhibitor treatment were excluded from the study. All available subjects were questioned for any type of urinary incontinence, either stress or urge type onset before hormonotherapy and only urinary continent women were included in the study. Hormone receptor-positive early-stage breast cancer patients were included if they were on tamoxifen as adjuvant hormonal therapy. Control group consisted of breast cancer patients who had never taken hormonal therapy. From those patients on tamoxifen, patients with a complaint of urinary incontinence that had started during tamoxifen usage were evaluated with Urogenital Distress Inventory Short Form (UDI-6), Incontinence Impact Questionnaire Short Form (IIQ-7) and Incontinence Quality of Life Questionnaire (I-QOL).

Statistical analysis

Data were analyzed using Statistical Package for the Social Sciences Version 20.0 (IBM Corp., Armonk, NY, USA). Continuous data were given as mean ± standard deviation. Categorical data were given as a percentage (%). Kolmogorov Smirnov test was used for normal distribution of data. The Mann-Whitney U test was used to compare two groups of data that did not conform to normal distribution among the groups; Student's t-test was used to compare normal distribution data. Chi-Square test was used to compare frequency data between two groups. A p-value <0.05 was considered statistically significant.

## Results

Seventy-four estrogen receptor-positive and 63 hormone receptor-negative early-stage breast cancer patients were included in this study. The demographic characteristics of the patients are shown in Table 1. The median age was 44 (30-65) years for tamoxifen users and 49 (27-64) years for the control group (p:0.061). The stages of the patients were similar for both groups (p:0.952). In the control group, 49.2% of the patients were premenopausal, whereas it was 78.4% in the tamoxifen group and the difference was significant (p<0.001). Since body mass index and parity are important factors for the occurrence of urinary incontinence, they were also evaluated for the two groups; however, the differences were statistically insignificant (p values were 0.563 and 0.755 respectively). Median tamoxifen usage time: 24 months (6-84)

**Table 1:** Demographic variables BMI: body mass index; DM: diabetes mellitus; HT: hypertension.

	Tamoxifen group (n=74)	Control group (n=63)	P
Age (y) (median)	44 (30-65)	49 (27-64)	0,061
Stage, n (%)			0,952
I	18 (24,3)	15 (23,8)	
II	31 (41,9)	28 (44,4)	
III	25 (33,8)	20 (31,7)	
Chemotherapy usage, n (%)	59 (79,7)	51 (81,0)	0,858
Menopausal status, n (%)			<0,001
Premenopausal	58 (78,4)	31 (49,2)	
Postmenopausal	7 (9,5)	29 (46,0)	
Perimenopausal	9 (12,2)	3 (4,8)	
Parity (median)	2 (0-6)	2 (0-8)	0,755
BMI (kg/m²)	27,80 ± 4,47	27,38 ± 3,90	0,563
Smoking (n (%))			0,224
Smoker	12 (16,2)	6 (9,5)	
Non-smoker	45 (60,8)	47 (74,6)	
Ex-smoker	17 (23,0)	10 (15,9)	
DM (n (%))	7 (9,5)	5 (7,9)	0,753
HT (n (%))	5 (6,8)	6 (9,5)	0,553

The number of patients with the complaint of urinary incontinence was 39 (52.7%) in the tamoxifen group and 5 (7.9%) in the control group (p<0.001). These 44 patients with the complaint of incontinence were evaluated with self-reported UDI6, IIQ7 and IQL forms and the scores were similar in between tamoxifen users and control group (p values were 0.118; 0.067 and 0.084 respectively) (Table 2).

**Table 2:** The questionnaire scores of the patients with the complaint of urinary incontinence UDI-6: Urogenital Distress Inventory Short Form; IIQ-7: Incontinence Impact Questionnaire Short Form; I-QOL: Incontinence Quality of Life Questionnaire.

Scores	Tamoxifen (n=39)	Control (n=5)	P
UDI6	20 ± 11	12 ± 7	0,118
IIQ7	23 ± 20	8 ± 10	0,067
IQL	92 ± 16	99 ± 4	0,084

In the multivariate analysis, there was no relation in between the presence of urinary incontinence and age, menopausal status, stages of patients, presence of diabetes mellitus, hypertension, parity, whether or not having chemotherapy. Although there was a strong relation of urinary incontinence with tamoxifen usage, the duration of treatment was not found to be associated with increased symptoms. Only there was a statistically significant difference detected for cigarette smoking and presence of urinary incontinence as 50% of those with incontinence were smokers; whereas, 24.7% of those without incontinence were smokers (p:0.003).

## Discussion

In this cross-sectional study, we have found that there was a statistically significant correlation between tamoxifen usage and the presence of urinary incontinence. The difference was not directly related to either menopausal status, age, presence of comorbid diseases or the duration of tamoxifen usage.

The effects of selective estrogen receptor modulators (SERMs) on the urethral epithelium of castrated female rats were evaluated in two different studies and hormonal therapy was found to reduce/reverse the incidence of urinary incontinence. In one of these studies, urethral thickness and weight were measured 30 days after the exposure of Tamoxifen and a significant increase in both the mean thickness and the mean weight of urethra was observed compared with the control rats (p<0.001) [11]. In the second study, a significantly increased distal urethral epithelial thickness was observed in castrated female rats given raloxifene [12]. Also in the in vitro cell culture models it was shown that estrogen and SERMs may help reduction of urinary incontinence by different mechanisms, either increasing collagen production in the extracellular matrix or increasing the muscle bundles [13-14]. However in another in vitro model, it was postulated that estrogen and raloxifene can cause urinary incontinence by reducing urethral resistance through a decrease in the expression of molecules that take part in the Rho-kinase signaling pathway [15]. There are conflicting data in these studies and the clinical relevance of these experimental results are still not clear.

In the study investigating hormone-related symptoms in a sample of 803 breast cancer survivors, it was reported that urinary incontinence complaints and scale was not significantly correlated with tamoxifen use, however, the severity was found to be worse in postmenopausal compared with premenopausal women [16].

In a population-based cross-sectional study, Baumgart et al. have evaluated 97 postmenopausal breast cancer patients on adjuvant endocrine treatment (47 of them on tamoxifen) and 105 age-matched postmenopausal control subjects for the prevalence and degree of urogenital symptoms. IIQ-7 and UDI-6 forms were used for urinary incontinence evaluation. It was reported that there was no difference in urinary incontinence symptoms between tamoxifen users, aromatase inhibitor users and control groups with or without estrogen treatments. The frequency of incontinence symptoms was between 54.4% and 67.6% in the groups that is higher than our results especially for the control group (it was found as 7.9% in our study) [17]. All of the patients were postmenopausal in the study of Baumgart et al. different from our study however we have found that the difference was independent from menopausal status.

In the study by Landi et al., self-reported urogenital symptoms data from 468 premenopausal and postmenopausal breast cancer patients, of whom 49% were receiving hormonal therapy either tamoxifen or aromatase inhibitors, were examined. Totally 18.4% of participants reported urinary incontinence, lower than reported prevalences in our study and the literatüre [18-19]. When compared with those breast cancer patients not using hormonotherapy, there was no significant association detected between urinary incontinence and endocrine therapy use either overall or tamoxifen or aromatase inhibitör [20]. 

In the case reported by Hasanov et al., a 67-year-old postmenopausal female patient receiving tamoxifen and having complaints of urinary incontinence was assessed by two questionnaires (IIQ-7 and UDI-7). The results revealed that she had severe incontinence. Two weeks after the cessation of tamoxifen the complaints completely resolved. When the hormone therapy was administered again the complaints were found to arise similarly supporting the worsening effect of tamoxifen on urinary symptoms as in our study [21].

Our study has some limitations. One is the limited sample size and consequently the lack of power for some statistical analyses. Since the number of patients with the complaint of incontinence was small, the insignificant results of the comparison of short questionnaire forms between groups may not be reflecting the real results. Another limitation is that the prevalence of urinary incontinence in control groups has been found to be quite different between studies in the literature and in our study. In some studies, healthy control was used but in this study, breast cancer patients not using hormone therapy was chosen as the control group and the complaint of urinary incontinence was only present in 7.9% of them, a significantly lower rate compared to most of the other studies.

## Conclusions

In this study, we have found that tamoxifen usage is correlated with urinary incontinence in early-stage breast cancer patients. The majority of women with a diagnosis of especially hormone receptor-positive early-stage breast cancer are expected to be long-term survivors and the impact of side effects of any treatment that may affect the quality of life should be examined, studied and enlightened more extensively. Further controlled studies on larger number of patients and controls are warranted for the effect of tamoxifen on urinary incontinence in the era of extended hormone therapies. Particular attention should be directed in future studies to compare different SERM types and urinary incontinence subtypes (stress incontinence and urge incontinence).
